# Challenges of status epilepticus management in a resource-limited setting: A review

**DOI:** 10.1016/j.ibneur.2024.06.001

**Published:** 2024-06-13

**Authors:** Kheng-Seang Lim, Si-Lei Fong, Siti Nasrina Yahaya, Minh-An Thuy Le, Herlyani Khosama

**Affiliations:** a Division of Neurology, Department of Medicine, Faculty of Medicine, Universiti Malaya, Malaysia; b Emergency and Trauma Department, Hospital Putrajaya, Malaysia; c Department of Neurology, University of Medicine and Pharmacy at Ho Chi Minh City, Ho Chi Minh City, Viet Nam; d Department of Neurology, Nguyen Tri Phuong Hospital, Ho Chi Minh City, Viet Nam; e Neurology Department, Faculty of Medicine, University Sam Ratulangi, Manado, Indonesia

**Keywords:** Status epilepticus, Resource-limited setting, Midazolam, Sodium valproate, Levetiracetam

## Abstract

Status epilepticus (SE) is a life-threatening neurological condition with significant mortality. Rapid management is essential to minimize the mortality and disability of SE. Two recent trials provided evidence to guide SE management in early and established stages. The Rapid Anticonvulsant Medication Prior To Arrival Trial (RAMPART, 2011) showed that intramuscular midazolam is a better alternative for early convulsive SE in prehospital settings. The Established Status Epilepticus Treatment Trial (ESETT, 2020) supported the use of sodium valproate and levetiracetam as second-line treatment for its efficacy and shorter administration time. However, there are challenges to revising the status epilepticus management in resource-limited settings, in pre-hospital, first- and second-line treatment, as well as management of refractory and super-refractory SE. These challenges included restrictions or lack of training in the administration of benzodiazepine in the prehospital setting, limited availability and accessibility of newer antiseizure medications (ASMs) in emergency departments and smaller hospitals, and low clinicians’ awareness of the latest evidence. A collaborative effort to educate, improve awareness, and make certain ASMs more readily available is recommended to achieve a better clinical outcome in SE.

## Introduction

Status epilepticus (SE) is a life-threatening neurological condition with significant mortality, and the outcome of SE is closely related to the seizure duration (Towne et al., 1994). Rapid management is essential to minimize the mortality and disability of SE. Many recent clinical trials had attempted to improve SE management in early, established, or refractory stages. In prehospital settings, a recent Rapid Anticonvulsant Medication Prior To Arrival Trial (RAMPART) proposed intramuscular midazolam as a better option for early convulsive SE (Silbergleit et al., 2011, Silbergleit et al., 2013, Silbergleit et al., 2012). The Established Status Epilepticus Treatment Trial (ESETT) supported the use of alternative second-line antiseizure medications (ASMs) besides phenytoin (Chamberlain et al., 2020, Cock and Group, 2011, Kapur et al., 2019). These led to updates in the evidence-based guidelines for SE management in developed countries (Anon, Glauser et al., 2016). However, the application of these findings in resource-limited countries is logistically and financially challenging. In a systematic review, SE is one of the most common causes of direct epilepsy-related deaths, which accounts for 13 % in low and middle-income countries (Levira et al., 2017). In lower-resource countries, practice in SE management remains the same (Lim et al., 2023). This review discussed the challenges of SE management in a resource-limited setting and proposed recommendations that complement the realities in resource-limited countries.

## RAMPART and ESETT Trials

The RAMPART (2011) was a double-blinded randomized clinical trial to determine the efficacy of intramuscular (IM) midazolam as non-inferior to intravenous (IV) lorazepam in adults and children patients treated for status epilepticus (SE). Adults and children ≥ 40 kg in the study were randomized to 10 mg IM midazolam or 4 mg IV lorazepam (Silbergleit et al., 2011). Treatment with IM midazolam was more likely to stop the seizure at emergency department arrival, without Emergency Medical Service (EMS) rescue therapy, hospitalization or admission to an intensive care unit, as compared with IV lorazepam (Towne et al., 1994, Silbergleit et al., 2013, Silbergleit et al., 2012). The seizure termination time after IV lorazepam was shorter but the time to administer IM midazolam was significantly shorter and easier, especially in those with difficult IV access. The overall interval time until seizure termination was similar in both IV and IM groups (Silbergleit et al., 2013).

The RAMPART study showed the superiority of IM midazolam over IV lorazepam and this result indicated that the best choice for prehospital treatment of SE by paramedics is IM midazolam. IV lorazepam may be the preferred initial treatment for SE in the emergency department, but it has a few limitations that make it less preferable for use by EMS. Lorazepam’s short shelf life out of refrigeration is not practical for EMS usage. IM midazolam has known water-soluble properties with excellent absorption and tolerability and can be more rapidly administered and absorbed than diazepam or lorazepam, leading to better clinical outcomes and lower rates of hospitalization and ICU admission (Silbergleit et al., 2012, Towne and DeLorenzo, 1999).

The ESETT was an investigator-initiated, multicenter, randomized, blinded, comparative-effectiveness trial to determine whether valproate or levetiracetam was superior in efficacy, safety, or cost-effectiveness in comparison to the current standard fosphenytoin for the treatment of convulsive SE ongoing or recurring despite adequate first-line benzodiazepines in the emergency department. The doses used in this study were higher, i.e., 40 mg/kg valproate, 20 mg/kg fosphenytoin, and up to 60 mg/kg levetiracetam over a shorter period of administration (10 minutes). The study was stopped early because it met a predefined futility criterion in the interim analysis. (Cock and Group, 2011, Kapur et al., 2019)

The ESETT study found that levetiracetam, fosphenytoin, and valproate each led to seizure termination and improved alertness by 60 minutes in approximately half the patients who have benzodiazepine-refractory convulsive status epilepticus (47 % with levetiracetam, 45 % with fosphenytoin and 46 % with valproate) (Kapur et al., 2019). The Valproate group had the shortest median time from the start of trial-drug administration to seizure termination (7.0 min), followed by levetiracetam (10.5 minutes) and fosphenytoin (11.7 minutes). The percentage of patients with recurrence of seizure activity between 60 minutes and 12 hours after the administration of trial-drug that needed more ASMs was 10.7 %, 11.2 %, and 11.2 %, respectively (Kapur et al., 2019).

## Prehospital treatment

Prehospital care is provided outside any healthcare facility, including initial assessment and treatment from the onset of the illness to the place of definitive treatment (Chew and Chan, 2011). In Malaysia, the prehospital care team is formed by the paramedics and consists of government health facilities, which can be government hospitals, teaching hospitals, or health clinics. Paramedic personnel are accredited to provide emergency care as outlined by the protocols outlined by the Malaysia Emergency Medical and Trauma Services, (Hisamuddin et al., 2007) and are trained to administer diazepam and midazolam in status epilepticus once they have completed Prehospital Care Level 1 and Level 2 training. Similarly, in Indonesia, ambulance services with trained paramedic personnel are accessible in certain regions, but benzodiazepine is only available in ambulances from the government or private hospitals. Whereas, in Vietnam, IV diazepam and IM midazolam are used in prehospital settings. There are also responders attached to private healthcare facilities or non-governmental organizations in Malaysia, such as the Malaysia Red Crescent Society, who do not necessarily have a paramedic training background but rather some formal first aid training such as Basic Life Support (BLS) certification. Thus, benzodiazepine is not readily accessible in ambulances from these private healthcare institutions or non-governmental institutions due to a lack of authorization, supervision, and training.

The initial management at the prehospital care level for early status epilepticus management, as outlined by the Malaysia guideline, is either buccal or intramuscular (IM) midazolam at 0.2 mg/kg or rectal diazepam with different dose recommendations according to age (Emergency Treatment of Epilepsy, 2017). IV Diazepam may also be considered as the first-line treatment if intravenous access is available because IV lorazepam is not available in many countries. In the PHTSE study, marked hypotension and respiratory arrest occurred more in the placebo than in the benzodiazepine (IV lorazepam and diazepam) group (Lowenstein et al., 2001). However, given the difficulty in getting intravenous access, rectal diazepam is often used. Rectal Diazepam is easier to administer with a readily prepared tube of 5 mg, whereas IV Diazepam or IM Midazolam needs its ampoule to be broken for usage. However, it is inconvenient because the patient needs to be undressed, which may not be socially acceptable, especially in an open environment. The next preferred option is IM midazolam, but the use is limited because it requires online medical directives from an Emergency Physician. To date, no studies have been conducted on primary care services, especially regarding their ability to provide first-line treatment. For government-based health clinics, the type of benzodiazepine available is rectal and IV diazepam. Some government health clinics, such as in Sabah, are equipped with Midazolam due to geographical limitations, with the intention of its utilization in the process of Rapid Sequence Intubation (RSI) during emergency intubation procedures. As there are no emergency physicians in government health clinic facilities, who can give online medical directives, the usage of IM Midazolam is not an option. Among private general practitioners, there is a lack of standardization in providing sufficient rescue treatment for patients with status epilepticus. In addition, the lack of awareness among general practitioners about calling the national emergency contact number “999”, which will trigger an ambulance response from the government healthcare facilities, may also cause a treatment delay.

## First-line hospital treatment

Benzodiazepine underdosing is not uncommon in Malaysia. Similarly, in Vietnam, underdosing is also a challenge because there is no national guideline for first-line treatment. For instance, 5 mg rectal diazepam administered to a 50 kg adult, equivalent to 0.1 mg/kg, is underdosing based on the recommended dose of 0.2 mg/kg for seizure cessation activity. This may contribute to poorer or partial seizure control, resulting in an unfavorable outcome. There is a common concern among clinicians that benzodiazepine overdosing may impose complications such as hypotension and respiratory arrest, but studies have proven that an appropriate dosage of benzodiazepine is safe to be used and will result in an early cessation of the seizure (Silbergleit et al., 2011, Lowenstein et al., 2001).

## Second-line treatment

Second-line treatment in SE can also be challenging in resource-limited settings. In our recent study, IV phenytoin remained the main second-line ASM across all age groups despite its longer administration time (20–25 minutes with a rate of 50 mg/min) and poorer seizure control rate as compared with IV sodium valproate, levetiracetam, or fosphenytoin that can be given over 10 minutes (Chamberlain et al., 2020, Alvarez et al., 2011). In addition, phenytoin also has the risk of worsening certain epileptic syndromes, such as Dravet syndrome among children and cardiovascular risk among the elderly. IV fosphenytoin is not available in many countries. Meanwhile, IV sodium valproate and levetiracetam are registered but are not available or readily accessible in most district hospitals in Malaysia (Fong et al., 2023). The cost of the drugs and the unfamiliarity of the non-neurologists with the drugs could be contributing factors, and underdosing could also be an issue. In Viet Nam, IV phenytoin, levetiracetam, and fosphenytoin are not available. IV sodium valproate is available only in a few central hospitals. In regional hospitals, the physicians would bypass the second-line treatment due to unavailability and use the anesthetic drugs in established SE.

## Refractory and Super-refractory SE

Refractory SE requires the use of anesthetic agents such as midazolam and propofol, which are widely available in most hospitals (Maheshwari et al., 2018). However, in certain types of SE, such as focal SE with preserved awareness (previously known as epilepsia partialis continua), nonconvulsive SE in the elderly, or absence SE, anesthetic agents with intubation can be avoided if other non-sedative ASMs are available. These include topiramate, zonisamide, lacosamide and perampanel. However, they are not available in most district and tertiary hospitals in Malaysia (Lim et al., 2023). Echoing the findings from the previous study that the availability of most second and third-generation ASMs differ among countries with different income levels (Pironi et al., 2022), our recent international survey found that these newer ASMs were not available or unaffordable across Asian lower-middle-income countries (Fong et al., 2023).

Super refractory SE (SRSE) is defined as SE that persists despite 24-hour treatment with an intravenous anesthetic agent or recurs after weaning off the anesthetic (Shorvon and Ferlisi, 2011). When resources are limited, referral of a case with SRSE to the nearest hospital with a neurologist for treatment escalation is necessary but may be unsafe if the patient’s state is critical and the transfer distance is long. Even in tertiary hospitals, more advanced treatment options for SRSE, such as immunotherapies, ketogenic diet (KD), electroconvulsive therapies (ECT), and hypothermia, were not always available (Lim et al., 2023). With the emergence of autoimmune origin of new-onset refractory status epilepticus (NORSE), early initiation of immunotherapies such as glucocorticoids, intravenous immunoglobulin (IVIG), plasma exchange (TPE), rituximab, tocilizumab, and anakinra are essentials to improve the seizure outcome (Wickström et al., 2022). However, the use of these treatments in resource-limited settings is limited by cost; for example, a single course of IVIG and TPE in India costs USD 4289 and USD 2040, respectively (Maheshwari et al., 2018).

## Recommendation

Several recommendations can be drawn to improve the care quality of SE in resource-limited settings. These strategies need to be implemented with support from the corresponding stakeholders and policymakers. Including the following strategies in the local practice guidelines will help improve awareness and improve the gaps in SE management from prehospital care to the SRSE stage.

1. Prehospital treatment and first-line treatment in the hospital: Early and adequate doses of benzodiazepine should be given, especially via non-IV routes such as IM midazolam.
2. Second-line ASMs: IV sodium valproate or levetiracetam should be made available in all secondary healthcare facilities, and the doses of these second-line ASMs should be emphasized in local guidelines to avoid under-dosing.
3. Refractory and super refractory SE: Early anticipation and referral to a neurologist should be advocated for early consideration and aggressive administration of other non-pharmacological treatment options or immunotherapies when indicated.
4. A collaborative effort between neurologists and emergency physicians is necessary to establish a standardized national guideline to enable early administration of IM midazolam in prehospital settings.
5. Continuing medical education among paramedics, emergency physicians, internal medicine specialists and neurologists is required to maintain the competency for early aggressive management of SE and acquire new knowledge in the developing field of SE.

## Conclusion

Despite recent evidence, there are challenges to updating the management of status epilepticus in all stages in resource-limited settings. These challenges included restrictions or lack of training in the administration of benzodiazepine, limited availability and accessibility of newer ASMs, and low clinicians’ awareness of the latest evidence. A collaborative effort to educate, improve awareness and make certain ASMs more readily available is recommended to achieve a better clinical outcome in SE.

## Compliance with ethical standards

Yes

## CRediT authorship contribution statement

**Kheng Seang Lim:** Conceptualization, Formal analysis, Methodology, Project administration, Supervision, Validation, Writing – original draft, Writing – review & editing. **Si-Lei Fong:** Conceptualization, Writing – original draft, Writing – review & editing. **Herlyani Khosama:** Conceptualization, Writing – original draft, Writing – review & editing. **Siti Nasrina Yahaya:** Conceptualization, Writing – original draft, Writing – review & editing. **Minh-An Thuy Le:** Conceptualization, Writing – original draft, Writing – review & editing.

## Declaration of Competing Interest

None
